# Investigating the correlation between paediatric stride interval persistence and gross energy expenditure

**DOI:** 10.1186/1756-0500-3-47

**Published:** 2010-02-26

**Authors:** Jillian A Fairley, Ervin Sejdić, Tom Chau

**Affiliations:** 1 Bloorview Research Institute, Bloorview Kids Rehab, Toronto, Ontario, Canada; 2 Institute of Biomaterials and Biomedical Engineering, University of Toronto, Toronto, Ontario, Canada

## Abstract

**Background:**

Stride interval persistence, a term used to describe the correlation structure of stride interval time series, is thought to provide insight into neuromotor control, though its exact clinical meaning has not yet been realized. Since human locomotion is shaped by energy efficient movements, it has been hypothesized that stride interval dynamics and energy expenditure may be inherently tied, both having demonstrated similar sensitivities to age, disease, and pace-constrained walking.

**Findings:**

This study tested for correlations between stride interval persistence and measures of energy expenditure including mass-specific gross oxygen consumption per minute (), mass-specific gross oxygen cost per meter (*VO*_2_) and heart rate (HR). Metabolic and stride interval data were collected from 30 asymptomatic children who completed one 10-minute walking trial under each of the following conditions: (i) overground walking, (ii) hands-free treadmill walking, and (iii) handrail-supported treadmill walking. Stride interval persistence was not significantly correlated with  (p > 0.32), *VO*_2 _(p > 0.18) or HR (p > 0.56).

**Conclusions:**

No simple linear dependence exists between stride interval persistence and measures of gross energy expenditure in asymptomatic children when walking overground and on a treadmill.

## Background

The human stride interval (i.e., the time between consecutive heel strikes of the same foot) exhibits statistical persistence, with correlations extending over thousands of strides [1]. This persistence is typically quantified in terms of *α*, a scaling estimate provided by detrended fluctuation analysis (DFA) [1,2]. To date, *α *has been found to change across the age spectrum, when certain neuromuscular disorders exist, and during some pace-constrained walking tasks [2-4].

Interestingly, physiological cost has demonstrated similar sensitivities to age, disease, and tempo-constrained locomotion with increased energy requirements having been linked to advanced ageing [5], pathology [6], and forced-rate stepping [7]. Given that stride interval dynamics are thought to reflect a certain extent of locomotor control [2], and that human locomotion is shaped by a preference for energy efficient movements [8], we hypothesized that energy and scaling measures would exhibit a certain degree of dependence. Specifically, we expected that decreased stride interval persistence would be associated with increased energy expenditure and vice versa.

The first study to investigate this dependence found no association in both adult and elderly populations [5]. However, stride interval persistence was quantified using time series obtained from just six minutes of walking, a time-frame requiring three repeated measures to provide a reliable estimate of persistence [9]. In this study, we assessed the correlation between stride interval persistence and gross energy expenditure in a paediatric population. Distinct from Malatesta et al. [5], we utilized longer (10-minute) time series to provide a more reliable quantification of stride interval persistence. Furthermore, in addition to unsupported treadmill walking, we considered overground walking and handrail-supported treadmill walking. These walking conditions, commonly implemented in gait research, have been found to differ in terms of both physiological cost [10,11] and stride interval persistence [12](Fairley, Sejdić and Chau: The effect of treadmill walking on the stride interval dynamics of children, submitted).

## Methods

### Experimental Protocol

The detailed experimental procedure is reported elsewhere (Fairley, Sejdić and Chau: The effect of treadmill walking on the stride interval dynamics of children, submitted). In brief, data were collected from 30 asymptomatic children (4-10 years old; 11 male) who completed one 10-minute walking trial under each of the following conditions: (i) overground walking, (ii) unsupported (hands-free) treadmill walking, and (iii) side-handrail supported treadmill walking. Mean (± SD) age, height, and body mass of subjects was 7.1 ± 1.6 years, 1.249 ± 0.115 m and 24.1 ± 4.9 kg, respectively. Subjects walked at their preferred speed, determined separately for each condition, and rested long enough before each trial to allow heart rate to return to pre-exercise resting values. All subjects abstained from eating or drinking anything other than water for at least two hours prior to data collection. This study was approved by the research ethics boards of Bloorview Kids Rehab and the University of Toronto and all subjects and their caregivers provided informed written assent and consent, respectively.

### Measurement Equipment

Stride interval time series were acquired from heel-strike data, obtained from force sensors (Model 406, Interlink Electronics, USA) on the subjects' shoes. Energy expenditure was measured, breath-by-breath, via a portable metabolic system (K4b^2^, Cosmed, Italy) that was carefully calibrated according to the manufacturer's instructions prior to each study session. Heart rate data were obtained via a heart rate transmitter (WearLink 31, Polar Electro, Finland). Two portable data collection units, secured to the subject's front and back via a waist harness, stored the data. The total mass of all subject-worn equipment was 2.5 kg. Additional equipment details are provided in (Fairley, Sejdić and Chau: The effect of treadmill walking on the stride interval dynamics of children, submitted).

### Data Analysis

Stride interval extraction and quantification of stride interval persistence was carried out as described in (Fairley, Sejdić and Chau: The effect of treadmill walking on the stride interval dynamics of children, submitted). Ultimately, DFA provided a scaling estimate, *α*, for each of the 180 stride interval time series (30 participants × 3 conditions × 2 feet). Since right and left foot stride interval persistence (*α*) was not significantly different (Fairley, Sejdić and Chau: The effect of treadmill walking on the stride interval dynamics of children, submitted), only right foot *α*-values were used in subsequent correlation analyses with measures of energy expenditure. For further analysis and discussion related specifically to the effect of walking condition and age on the *α*-values obtained in this investigation, the interested reader is referred to (Fairley, Sejdić and Chau: The effect of treadmill walking on the stride interval dynamics of children, submitted).

Energy expenditure was assessed in terms mass-specific gross oxygen consumption per minute (; ml·kg^-1^·min^-1^), mass-specific gross oxygen cost per meter (*VO*_2_; ml·kg^-1^·m^-1^), and heart rate (HR; bpm). These parameters were each computed, as an average of the recorded breath-by-breath values, over the last three minutes of each walking trial. With subjects having walked for seven minutes prior, this time frame is considered sufficient to ensure that the child reached a steady-state of exercise [13]. Data points for which the respiratory exchange ratio exceeded 0.9 were discarded. This ratio is indicative of anaerobic activity and should not be expected during comfortably paced walking [6]. In total, 90 values were obtained for each energy measure (30 subjects × 3 conditions).

Prior to statistical analysis, all data were tested for normality using the Chi-squared goodness-of-fit test [14] to inform the choice between Pearson's (*r*) or Spearman's (*ρ*) correlation coefficients. Correlations were performed between right foot *α*-values and each measure of energy expenditure, first considering data from each walking condition separately, and then considering all walking conditions together.

## Results

**Figure 1:**
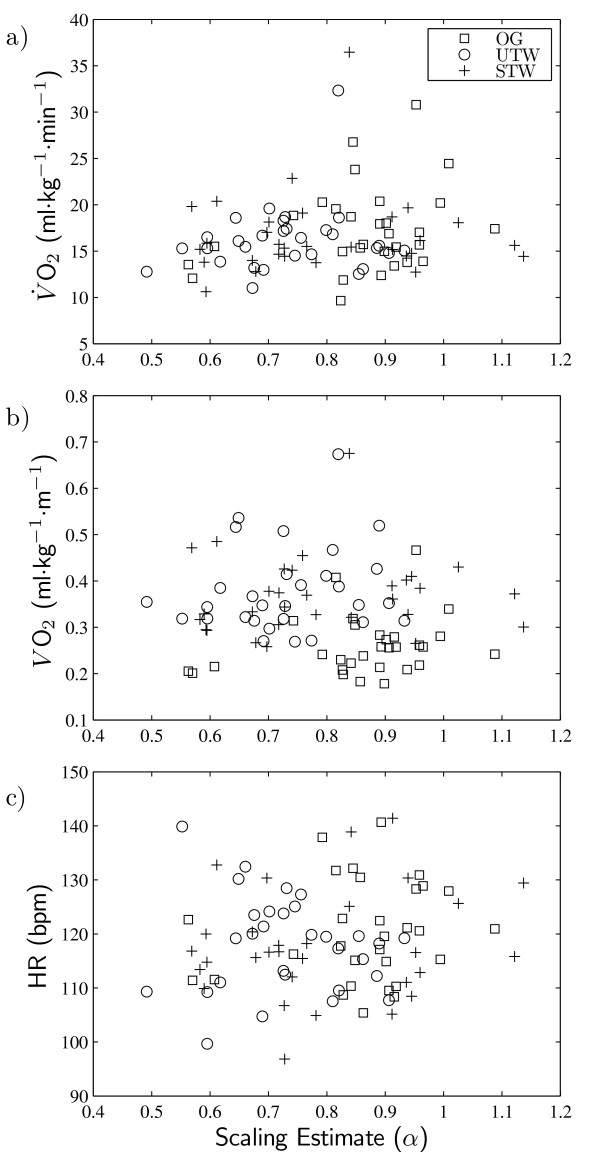
**Scatter plots of gross energy expenditure measures versus scaling estimate**. Scatter plots of (a) mass-specific gross oxygen consumption per minute (), (b) mass-specific gross oxygen cost per meter (*VO*_2_) and (c) heart rate (HR), all versus right foot scaling estimates (*α*). OW = overground walking; UTW = unsupported treadmill walking; STW = supported treadmill walking.

Considering each walking condition separately, right foot *α*-values were not significantly correlated with  (|*ρ*| < 0.188, p > 0.32), *VO*_2 _(|*r*| < 0.254, p > 0.18), or HR (|*ρ*| < 0.111, p > 0.56). Furthermore, there were no significant correlations between *α *and any of the measures of energy expenditure when all walking conditions were considered together (|*r*| or |*ρ*| < 0.144, p > 0.17). These results are depicted in Figure 1. The values of energy expenditure and *α *obtained in this study resonated closely with literature values reported for children of comparable age who performed similar walking tasks (e.g., [15-18]).

## Discussion

This paediatric study found no correlation between *α *and measures of gross energy expenditure. These results corroborate findings for adults and the elderly [5], and suggest that stride interval dynamics are not simply an outward indicator of gross physiological cost. Gross energy measures encompass basal metabolic rate and the energy required for maintenance of an upright body position, in addition to the metabolic demands of making the walking movements prescribed in this study [5]. Therefore, if *α *is indeed a reflection of neuromotor control [2](Fairley, Sejdić and Chau: The effect of treadmill walking on the stride interval dynamics of children, submitted), these gross energy measures may not be sensitive enough to reflect the underlying neurological processes associated with motor activity alone. Alternatively, given that an association between either energy utilization or dynamic stability has yet to be found [5](Fairley, Sejdić and Chau: The effect of treadmill walking on the stride interval dynamics of children, submitted; Chang, Sejdić, Wright and Chau: Measures of dynamic stability: detecting differences between walking overground and on a compliant surface, submitted), stride interval persistence may not simply imply biomechanical efficiency. To this end, recent conjecture proposes that *α *may be more directly related to the extent of cognitive processes required of a particular gait task [3](Fairley, Sejdić and Chau: The effect of treadmill walking on the stride interval dynamics of children, submitted). Thus, future research may include investigation into the association between *α *and net energy expenditure (i.e., the incremental cost of walking over resting) or the effect of dual task conditions (i.e., cognitive load) on *α*.

## Conclusions

This study did not identify any significant correlations between stride interval persistence and measures of gross energy expenditure in a paediatric sample, suggesting that no simple linear association exists. The physiological meaning of stride interval persistence remains to be identified before *α *can become a truly informative clinical tool.

## Competing interests

The authors declare that they have no competing interests.

## Authors' contributions

JAF designed the study, carried out the data collection and analysis, and drafted the manuscript. ES assisted with data analysis and manuscript revision. TC assisted with study design, data analysis and manuscript revision. All authors read and approved the final manuscript.
